# Cancer Stem Cells: Dynamic Entities in an Ever-Evolving Paradigm

**DOI:** 10.4172/0974-8369.1000S2-001

**Published:** 2014-11-11

**Authors:** Hernando Lopez-Bertoni, Yunqing Li, John Laterra

**Affiliations:** 1 Hugo W. Moser Research Institute at Kennedy Krieger, USA; 2 Johns Hopkins School of Medicine, Baltimore, Maryland, USA

## Abstract

The cancer stem cell (CSC) hypothesis postulates that there is a hierarchy of cellular differentiation within cancers and that the bulk population of tumor cells is derived from a relatively small population of multi-potent neoplastic stem-like cells (CSCs). This tumor-initiating cell population plays an important role in maintaining tumor growth through their unlimited self-renewal, therapeutic resistance, and capacity to propagate tumors through asymmetric cell division. Recent findings from multiple laboratories show that cancer progenitor cells have the capacity to de-differentiate and acquire a stem-like phenotype in response to either genetic manipulation or environmental cues. These findings suggest that CSCs and relatively differentiated progenitors coexist in dynamic equilibrium and are subject to bidirectional conversion. In this review, we discuss emerging concepts regarding the stem-like phenotype, its acquisition by cancer progenitor cells, and the molecular mechanisms involved. Understanding the dynamic equilibrium between CSCs and cancer progenitor cells is critical for the development of novel therapeutic strategies that focus on depleting tumors of their tumor-propagating cell population.

## Introduction

Physicians and scientists have wondered about the origins of tumors since the 18th century, when Giovanni Morgagni of Padua established autopsies as a routine procedure to determine why patients died. In 1863, the implementation of the microscope as a scientific tool lead Rudolf Virchow to speculate about the cellular origin of cancer when he observed that some tumor cells within pathological specimens seemed less differentiated than others, and in 1889 Stephen Paget proposed the seed-and-soil hypothesis, which provided an explanation for how tumors metastasize to distant sites. Discoveries in the early 1900’s by Peyton Rous and Theodore Boveri established that cancer can be caused by viruses and chromosomal abnormalities [1], laying the foundations for modern oncology and the study of cancer biology.

The concept of stem cells applied to tumor initiation has been around for quite some time. Building on the breakthroughs of Morgagni and Virchow, pathologists increasingly recognized the histopathological heterogeneity within solid tumors. Furth and Kahn in 1937 and then others, using serial transplantations and limiting dilutions, showed that a single cell was able to propagate tumor xenografts that recapitulate the features of the original clinical tumor [2–4]. Furthermore, Pierce demonstrated that cells isolated from teratocarcinomas were highly tumorigenic when in an undifferentiated state, but lost their ability to form tumors upon differentiation [5]. The cancer stem cell hypothesis really started to take shape as a result of this early work. Pierce conducted pioneering lineage tracing experiments and was able to show that labeled undifferentiated squamous cell carcinoma cells gave rise to fully differentiated cells. Again, the labeled differentiated cells lost the ability to form tumors [6]. These experiments provided strong evidence to support a hierarchical organization within tumors, leading Pierce to propose the Cancer Stem Cell (CSC) hypothesis [7]. Pierce’s findings changed the way physicians and scientists looked at cancer treatment and paved the road for the use of combinatorial therapies to treat tumors as well as highlighting the idea that targeting the “cell of origin” may be more efficacious than focusing therapy on the bulk population of the tumor cells [8].

Beginning in the late 1970’s, the emerging discoveries of tumor suppressing and tumor promoting genes and their mutations constituted a conceptual paradigm shift that relegated the CSC hypothesis to a supporting role. In 1976, Nowell proposed that tumors are derived from a single cell, and that tumor progression is the result of acquired mutations that give the tumor cells an advantage [9]. This view of “clonal evolution” of tumors was modeled in colon cancer [10] and seemed to provide an iron-clad explanation for how tumors come to be, how they evolve, and why they become more aggressive. It did, however, fail to fully explain why some tumors are highly heterogeneous in nature and did not provide a satisfying answer as to why tumors recur after prolonged periods of dormancy.

In the early 1990’s, groundbreaking work had been taking place in the area of hematopoietic stem cell research, namely the characterization of well-defined and validated surface markers that allowed for identification of undifferentiated cells as well as different lineages resulting from hematopoeitic stem cells [11–13]. In the mid-1990’s John Dick took advantage of this technology and studied heterogeneity within leukemia. His results showed that a subtype of acute myeloid leukemia (AML) was able to reconstitute tumors in immune-compromised mice, but most importantly, these cells were isolated from a specific fraction (i.e. CD34+CD38−). They further showed that only 1 out of 1,000,000 cells had the ability to form tumors [14–16]. These studies sparked a renewed interest in the CSC hypothesis, which lead to similar studies in solid tumors. Researchers showed that breast cancer and glioblastoma are made up of a heterogeneous population of tumor cells, but only a certain population of cells (CD44+CD24−/low for breast cancer and CD133+ for glioblastoma) formed tumors that could be serially passaged and had the ability to reconstitute the original tumor morphology [17]. Since then, cells with the capacity to reconstitute tumors after isolation using defined markers have been identified in several tumor types [18–20].

**Figure 1:**
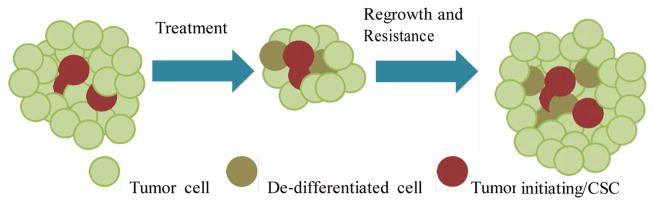
The CSC hypothesis postulates that there is a hierarchy of cellular differentiation within cancers and that the bulk population of tumor cells is derived from a relatively small population of multi-potent neoplastic stem-like cells (CSCs). These cells play a crucial role in tumor maintenance, therapeutic resistance, and tumor propagation. The CSC hypothesis predicts that targeting the CSCs will more effectively treat tumors and prevent recurrence.

The CSC hypothesis postulates that there is a hierarchy of cellular differentiation within cancers and that the bulk population of tumor cells is derived from a relatively small population of multi-potent neoplastic stem-like cells (CSCs) that play a particularly important role in maintaining tumor growth through their unlimited self-renewal, therapeutic resistance, and capacity to propagate tumors through asymmetric cell division [20–24] (Figure 1).

At a practical level, this hypothesis predicts that targeting the CSCs will more effectively treat tumors and prevent recurrence. Differentiation therapy has emerged as an approach to force tumor cells into lineages that are less efficient tumor propagators and more susceptible to treatment.

## The CSC Hypothesis: Where Do Tumor-Initiating Cells Come From

**Figure 2:**
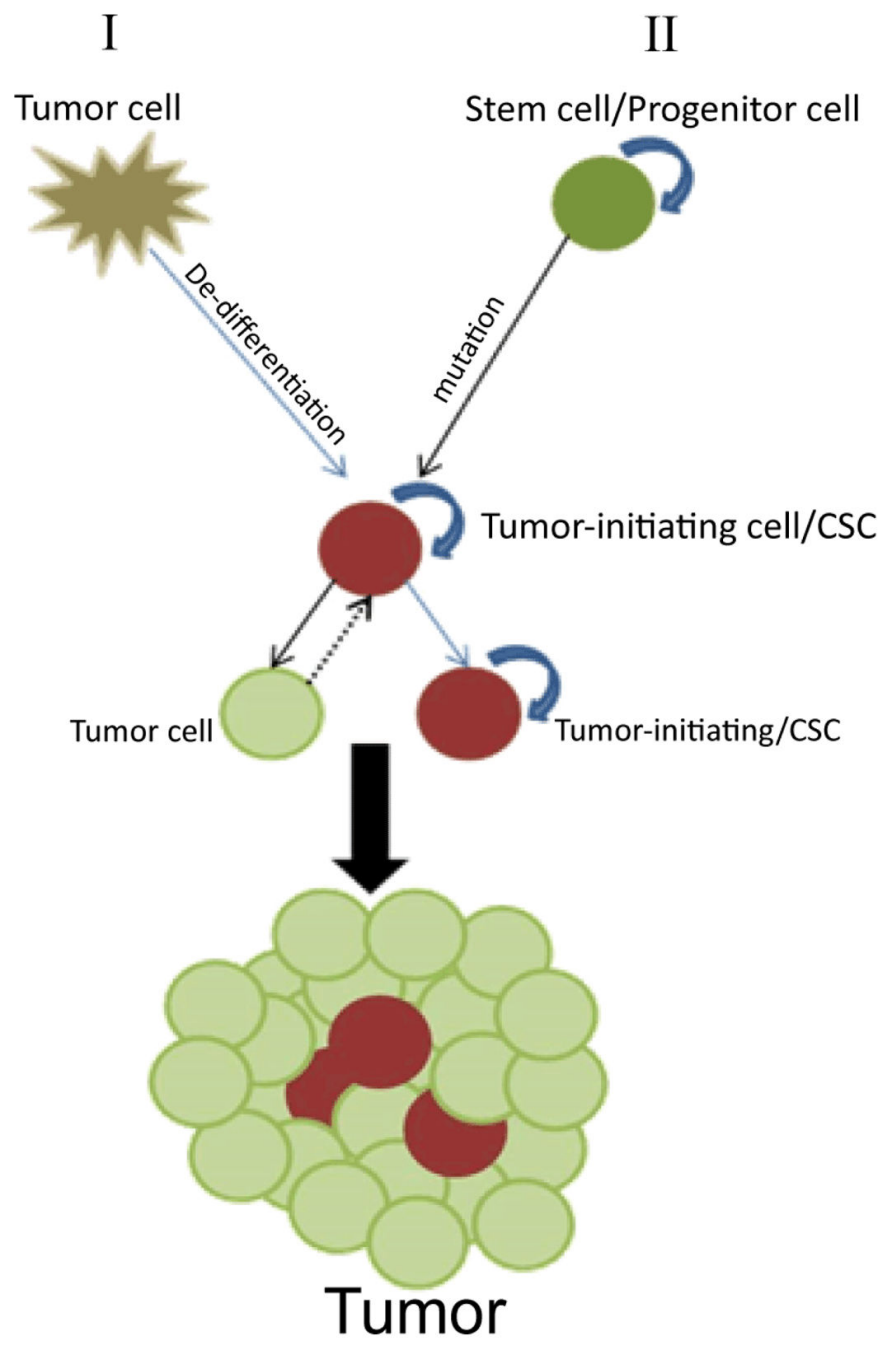
CSCs can arise in several manners: (i) from more differentiated neoplastic cells that in response to environmental cues or acquired mutations activate de-differentiation mechanisms; or (ii) in other cases, the CSC population arises from normal stem cells that acquire tumorigenic potential through mutations. A tumor that conforms to the CSC hypothesis should be heterogeneous and contain a subset of multipotent, relatively undifferentiated cells able to propagate tumors in transplantation models and generate more differentiated progenitor cells with limited tumor propagation potential.

As mentioned earlier, there is a growing body of evidence supporting the idea that the population of cells with stem-like potential exists in several types of cancer[13,14,16,17,19,20,25] and as few as one of these cells is sufficient to propagate tumor growth and recurrence [5]. Despite the fact that we can isolate these CSCs, we still do not understand their nature and origin. The CSC hypothesis draws parallels from the cellular hierarchies of normal tissue development, where undifferentiated multi-potent cells have the ability to differentiate and give rise to specialized cells that eventually constitute heterogeneous tissue. The stem cells serve to support tissue regeneration and injury repair. What the CSC hypothesis does not address is whether these cancer initiating cells are normal stem cells that have gained tumorigenic capabilities, or are tumorigenic cells that have gained a stem-like phenotype (Figure 2).

So rather than focusing on the cell of origin per se, the CSC hypothesis proposes a pragmatic approach and offers an “operational” definition of what constitutes a CSC. Regardless of the cell of origin, a tumor that conforms to the CSC hypothesis should be heterogeneous and contain a small population of multi-potent, relatively undifferentiated cells able to propagate tumors in transplantation models [26–29] and generate more differentiated progenitor cells with limited tumor propagation potential. Although the CSC hypothesis does not directly address the cell of origin issue, understanding how these tumor-initiating cells arise should provide significant insights. Paramount to cancer therapy is identifying cancer-specific characteristics of these cells that can be exploited for treatment to minimize effects on normal cells.

Great efforts have been put forward into understanding where CSCs come from. There are instances, such as intestinal cancers, where hierarchically organized tumors originate from normal cells [30,31]. In other cases, it appears that the CSC population arises from more differentiated neoplastic cells that respond to environmental cues or acquired mutations that activate de-differentiation mechanisms [32,33]. More recently, the idea that differentiated cells can revert to a more stem-like state and in doing so contribute to tumor formation, has added an extra level of complexity to the system [34–37].

### Epigenetic Reprogramming

**Figure 3:**
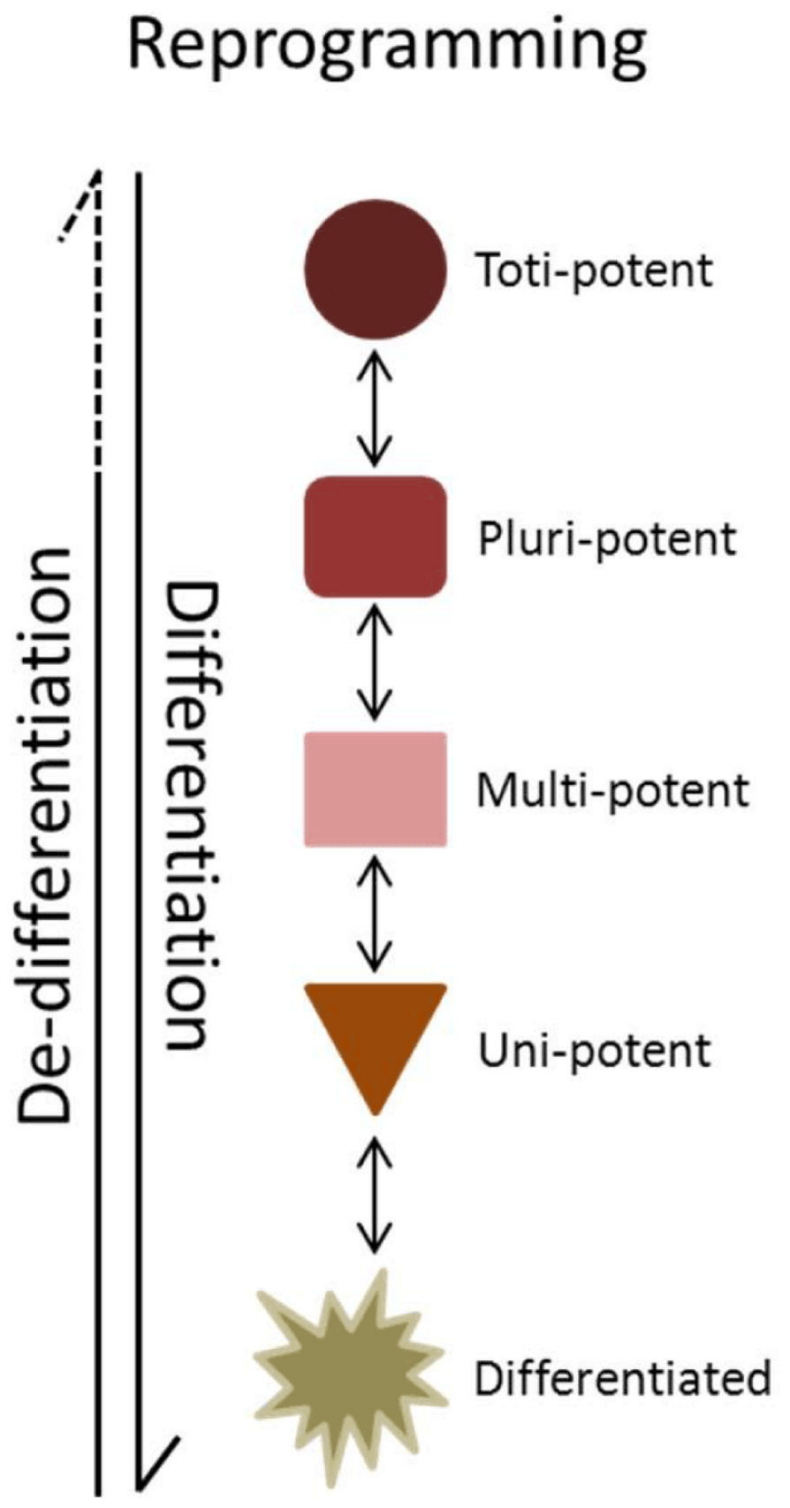
Reprogramming is a process during which cells either commit to a more differentiated state (i.e. differentiation), or, in the case of neoplastic disorders, revert to a more stem-like state (i.e. dedifferentiation).

**Figure 4:**
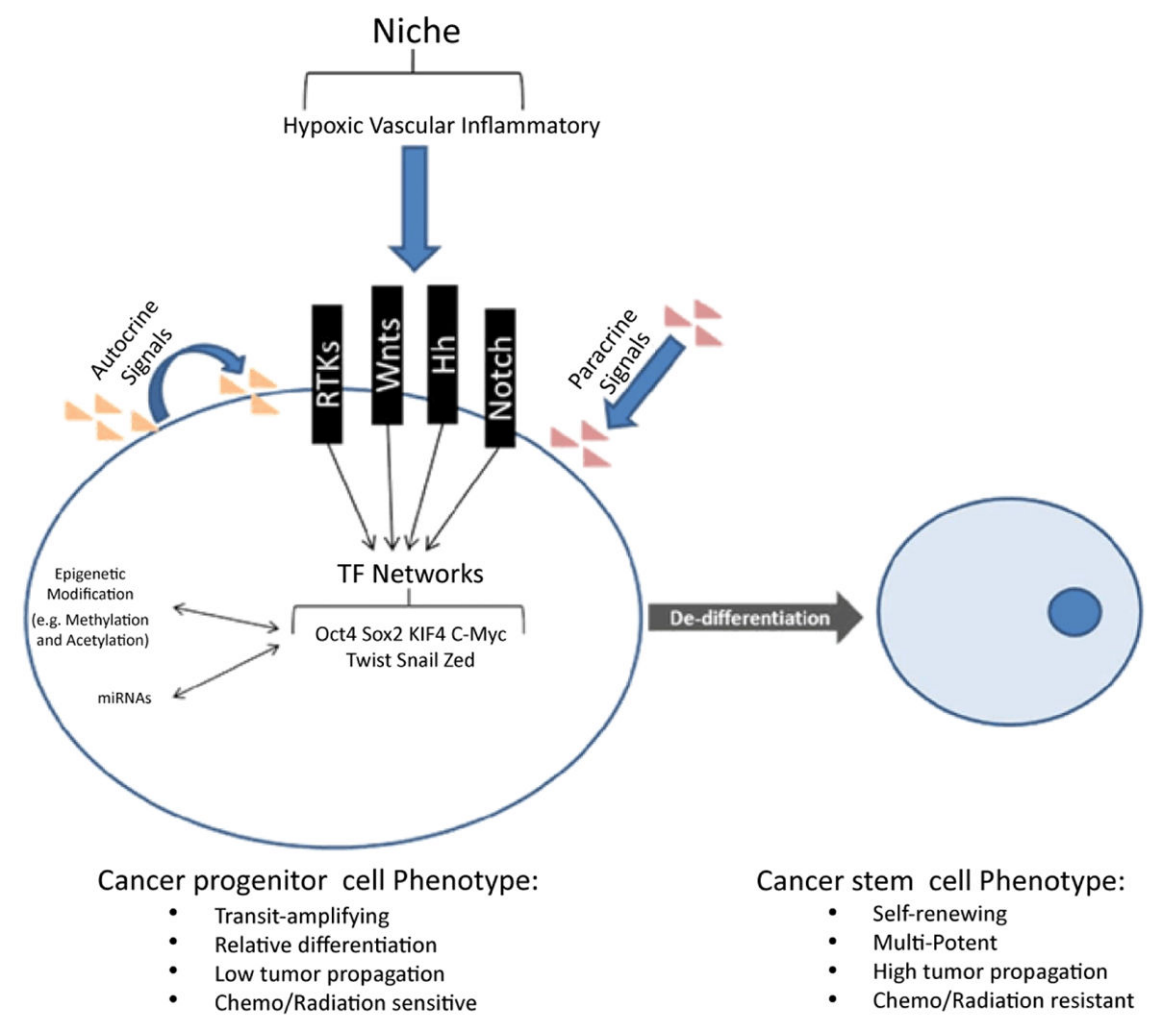
De-differentiation encompasses molecular changes that results in cells reverting to a more stem-like state. This process is influenced by autocrine and paracrine pathways including environmental cues that modify the DNA methylation landscape and histone marks, modulate the expression of transcription factors and regulate coding and non-coding RNAs all leading to fate-determining gene expression changes.

The term reprogramming, in the context of stem cell biology, is usually directly associated with the Yamanaka [38] experiments and the transition from fully differentiated cells to induced pluripotent stem cells (iPSC). Reprogramming is a complex and dynamic process that occurs in stages and is multi-directional (Figure 3). Each time a cell takes a step towards differentiation or de-differentiation, we envision a plethora of molecular events. These include modulating the expression of transcription factors, modifying the methylation landscape, adapting histone marks, and differentially regulating coding and non-coding RNA. It is, therefore, not surprising that transcriptional networks play important roles in maintaining stem cell self-renewal, cell lineage determination, and progenitor cell growth in normal as well as neoplastic tissues. It is now known that expression changes in defined sets of transcription factors are sufficient to drive cell reprogramming to different cell types [39,40]. Han et al. [40] showed that manipulating the transcription network in mouse fibroblasts generates induced neuronal stem cells (iNSC) that exhibit no differences in terms of morphology, gene expression, epigenetic features, differentiation potential, and self-renewing capacity, as well as in vitro and in vivo functionality when compared to wild type neuronal stem cells (Figure 4).

This study highlights how under certain conditions cells can be induced to undergo a complete change of identity. Although the authors of this study focused on the implications of this plasticity for regenerative medicine purposes, one can envision a similar process taking place in tumor cells. By displaying this inherent plasticity, tumor cells can adapt to multiple environments and dynamically alter their dependence on specific signaling and metabolic pathways, features associated with metastasis and therapeutic resistance [41].

As mentioned earlier, expression of a defined set of transcription factors is sufficient to reprogram mouse and human cells to an induced-pluripotent state. These iPS cells resemble embryonic stem cells since they possess the capacity to differentiate into all tissue subtypes [38]. In cancer, expression of these transcription factors (Oct4, Sox2, c-Myc, and Klf4) has been found to correlate with poor prognosis [42–44] and tumor progression[45–47].

The high similarity in gene expression profiles of embryonic stem cells (ESCs) and high grade tumors [48] further supports the molecular parallels between the stem cell phenotype, induced pluripotency, and cancer [48]. This suggests a de-differentiation mechanism whereby expression and function of reprogramming transcription factors influence the tumorigenic potential of cells by driving them to less differentiated and potentially more aggressive stem-like states. Indeed, expression of one or more of these reprogramming factors has been shown to switch tumor cells to a more stem-like state and resulted in a more aggressive tumor phenotype. For instance, expression of Oct4, Sox2, c-3 Myc, and Klf4 in gastrointestinal cancer cells results in a more ESC-like state [49]. Forced expression of Oct4 and Nanog caused lung cancer cells to express the stem cell marker CD133 and grow as spheres in defined medium similar to non-neoplastic stem cells, develop drug-resistance, and acquire enhanced tumor propagating capacity consistent with de-differentiation to a more stem-like state [45]. Recent findings from our laboratory and validated by others have directly linked the stem-like phenotype of GBM cells to the oncogenic receptor tyrosine kinase c-Met [37,50–52]. This work shows that c-Met marks GBM stem cells, induces the expression of reprogramming factors, including Oct4, Sox2 and Nanog and thereby dynamically regulates the degree of GBM cell stemness in vitro and in vivo[37,52]. These findings establish dynamically-regulated de-differentiation mechanisms involved in cancer stem-cell generation and maintenance.

Our findings that Nanog silencing inhibits the reprogramming capacity of c-Met signaling in GBM cells highlights Nanog’s emerging role in maintenance of CSCs [37]. These results are in line with those showing that Nanog serves as a gatekeeper to full de-differentiation [53]. More recently, Nanog has been shown to accelerate reprogramming and induce pluripotency of pre-iPS mouse cells [54]. Other studies show that Nanog induction is sufficient to not only induce de-differentiation of mouse astrocytes in the absence of p53, but also confers tumorigenic potential to these cells [55,56]. Interestingly, cancer vaccines have been found to induce Nanog expression resulting in a sub-set of cells that gain stem-like potential and become resistant to tumor-specific CTLs [57]. These studies stress the importance of understanding how reprogramming transcription factors contribute to the CSC phenotype and suggest that tumor cells can use reprogramming factor expression to their advantage in order to survive and propagate de-differentiation and acquisition of stem cell qualities results in tumor cells with self-renewal capability, tumor-propagating capacity, and treatment resistant. Additionally, they indicate that combining cytotoxic therapies with approaches that target the CSC population may be a particularly efficient way to treat cancer.

The evidence presented so far argues that neoplastic cells are inherently plastic and transcription factors play critical roles in tumor cell fate determination through the process of reprogramming. We also proposed the possibility of neoplastic cells taking advantage of these capabilities in order to promote and maintain the tumor. But is there evidence that tumor cells can hijack this mechanism under physiological conditions and use it to their advantage? Under stress conditions, several cell types have the ability to de-differentiate in order to supplement the stem cell population to support tissue repair [58–61].

Interestingly enough, it appears that tumor cells can take advantage of a similar process of “stress-induced reprogramming” and de-differentiate to a stem-like state with the capacity to maintain or even reconstitute a malignancy.

For instance, glioma stem cells (GSC) are thought to reside in a hypoxic niche. Hypoxia stabilizes HIF2, which in turn can activate stemness genes that contribute to tumor initiation by reprogramming cells through a de-differentiation mechanism [62–65]. As an example, this process may be activated by stress resulting from therapeutic intervention. Legadec et al. [34] found that radiation therapy can cause fully differentiated breast cancer cells to de-differentiate and become induced-breast cancer stem cells (iBCSC). These iBSCS re-express reprogramming factors Oct4, Sox2, Nanog, and Klf4 and this de-differentiation process was found to partially depend on Notch signaling. These studies convey the possibility that a fraction of tumor cells can de-differentiate in response to therapy and potentially contribute to recurrence and therapy resistance.

## Role of the Microenvironment in CSC Reprogramming and Maintenance

It is well-recognized that the microenvironment plays an important role in supporting stem cell populations within tissues. It is now well-established that discrete environmental niches exist (e.g. subventricular zone in the brain, perivascular niche, hypoxic niche) that provide molecular cues for maintaining stem-like states in selected cell populations [30,64,66–68]. As with normal stem cells, microenvironmental niches seem to play a crucial role in supporting the CSC population. Communication between CSC and niche appears to be bidirectional, and stem cells within tumors may even have the ability to modify their microenvironment with the effect of amplifying niche-derived signals that support and replenish the pool of tumor-initiating cells. Therefore, understanding the relationship between CSC and their microenvironment has great promise in terms of treating and managing the disease [66,69].

Hypoxia is a hallmark of the cancer microenvironment and plays an important role in inducing and maintaining neoplastic stem-like phenotypes in cancer [70]. The hypoxic environment of the tumor induces expression of HIF, which in turn activates reprogramming factors that drive cancer cell de-differentiation [62–65]. Studies in 11 different cancer cell lines found that HIF, when combined with core reprogramming transcription factors, enhances cell de-differentiation and tumor propagating potential [71]. Heddleston et al. [65] found that hypoxia promotes self-renewal of glioma stem cells (GSC) as well as non-stem cells through a process regulated by HIF2 and attribute this de-differentiation response to the increased expression of Oct4, Sox2, Nanog, and c-Myc. Many paracrine signals, in addition to hypoxia, have been found to influence tumor cell stemness. For instance, IL-6 secreted by tumor-infiltrating macrophages can increase the tumor-initiating capacity and drug resistance capability of neoplastic stem-like cell populations by inducing Stat3 and Hedgehog signaling. IL-6 was found to enhance the conversion of breast cancer progenitor cells to a more stem-like state via a positive feedback loop involving NF-kB, Lin28, and miRNA Let-7 [72]. Stromal cells influence the cancer stem-like phenotype of cells through paracrine signaling. In colorectal cancer, myofribroblasts in the tumor environment were found to secrete hepatocyte growth factor (HGF), which can support the stem cell population, at least in part, by activating Wnt signaling [73]. Relevant findings in our lab demonstrate that HGF/c-Met signaling drives brain CSC phenotype by inducing reprogramming transcription factors [37] and that inhibiting this axis in vivo depletes tumors of their tumor-initiating capacity [52]. These findings highlight that stem-like tumor-initiating cells are dynamically regulated by their microenvironment in vivo and that c-Met pathway inhibition can deplete tumors of their tumor-propagating stem-like cells [52].

The perivascular niche has been found to play a particular role in supporting the tumor-initiating cell population of brain tumors [74,75]. Callabrese et al. [67] found that endothelial cells interact closely with brain cancer stem cells and support the self-renewal capability of this cell population. This study found that endothelial cell and blood vessel numbers were directly proportional to the ability of tumor cells to propagate xenografts in mice. Brain CSCs respond to endothelial-derived nitric oxide with activation of Notch signaling, a known driver of cancer cell stemness. Cross talk between endothelial cells and tumor cells serves to maintain and expand the CSC pool. In cutaneous papilloma, CSCs have been found to preferentially localize within a perivascular niche where vascular-endothelial growth factor (VEGF) acts upon endothelial and tumor cell receptors to both induce angiogenesis and expand the cancer stem cell pool by stimulating symmetric cell division [68]. Understanding the bidirectional relationship between CSCs and their microenvironment niches should identify novel targets and strategiesfor therapeutic intervention.

## Contribution of Epigenetic Modifications to the Stem Cell Phenotype

The evidence presented so far strongly supports the function of Yamanaka transcription factors induced by oncogenic signaling and microenvironmental cues as “drivers” of cancer cell reprogramming and stemness. These fate-determining transcriptional events are constrained by promoter accessibility which is determined by the influences of histone modifications on chromatin architecture and by promoter DNA methylation [76].

DNA methylation is established by the de novo DNA methyltransferases (DNMTs) Dnmt3a and Dnmt3b, and is maintained through cell division by Dnmt1 [77] in a process involving the addition of methyl groups to cytosine residues [78,79]. Hypermethylation of tumor suppressor genes and de-methylation of oncogenes may play a role in tumor initiation and progression [80,81]. If these changes happen early enough during tumor formation, it is thought that neoplastic cells may even become addicted to these epigenetic changes [82]. Furthermore, changes in DNA methylation regulate genes involved in angiogenesis and metastasis [83]. Changes in methylation patterns are associated with the transition of stem cells from pluripotent to a more differentiated state [84]. DNA methylation is an important mechanism by which differentiation programs are silenced in stem cells as a pre-requisite to maintaining self-renewal and multi-potency [85–87]. Forced expression of Dnmt3b promotes tumorigenesis of colon cancer cells in vivo by silencing a specific set of tumor suppressor genes [88]. Other studies in glioma have associated high expression of Dnmt1 and Dnmt3b with hypermethylation of tumor suppressor genes that regulate genomic stability and cell cycle, and influence cell tumorigenicity [86]. Additionally, deregulation of DNMTs has been associated with the tumor cell phenotype and stem cell compartment in glioblastoma [87], linking DNMT deregulation with the tumor-initiating cell population. Interestingly, reprogramming factors (i.e. Oct4 and Nanog) can directly induce DNA methyltransferase expression (i.e. Dnmt1) and control the fate of mesenchymal stem cells [89]. However, whether specific DNA methylation signatures play a role in cancer stem-like phenotype acquisition and/or maintenance remains unclear. DNMT inhibitors have emerged as a promising option for treating cancer, especially in combination with other established approaches [90]. The use of 5-azacitidine (5-Aza) [91], a pan-DNA methyltransferase inhibitor, proved to be an effective way to treat AML [92] and it worked as an adjuvant in prostate cancer when combined with bicalutamide [93]. DNA methyltransferase inhibition can induce differentiation of stem cells [89,94]. Interestingly, DNMT inhibitors have been shown to enhance differentiation of leukemia-initiating cells [95] as well as hepatic cells [96]. Treatment of melanoma cells with decitabine, a Dnmt1 inhibitor, induced differentiation of these cells and inhibited tumor growth in vivo in a mouse melanoma model [97]. This evidence suggests that modulating DNA methylation can be an effective way of depleting the CSC population. These studies emphasize the need to better understand how changes in the methylation landscape contribute to the CSC population in order to target tumor-initiation and propagation more efficiently.

Many transcription factors bind DNA regions containing CpG sequences. It is thought that DNA methylation interferes with this process by changing the recognition sites at the cytosine residue. This process has been described for several transcription factors, including transcription factors with reprogramming capability [98–100]. These data support the current dogma wherein DNA methylation determines whether a transcription factor binds DNA or not. It has to be recognized, however, that there are some exceptions to the rule. For instance, the SP1 consensus sequence contains a CpG island and studies indicate that this transcription complex can bind DNA regardless of methylation status [101]. This may suggest that the methylation of some promoters modulates rather than shuts off target gene expression, thus offering a new and elegant way of regulating gene networks which could have important implication on reprogramming and other processes.

The nucleosome is a fundamental building block of chromatin, consisting of DNA tightly associated with histone proteins [102]. As mentioned earlier, chromatin architecture regulates accessibility of transcription factors to DNA and in doing so, controls several biological processes. Chromatin structure is regulated by epigenetic mechanisms including histone modifications, DNA methylation, histone variants, and nucleosome remodeling complexes [103]. Nucleosome positions are dynamic and correlate with gene expression changes and cell fate [104]. Histone modifications destabilize nucleosomes resulting in chromatin configurations that support transcription. For instance, tri-methylation of lysine 4 on histone3 (H3K4me3) and histone variant H2A.Z are associated with open chromatin structures amenable to gene transcription, and H3K36me3 is thought to positively regulate transcriptional elongation[105]. Histone marks have also been found to play a role in activating enhancer regions (e.g. H3K4me1 and H3K27ac) [106]. The same way open chromatin promotes gene transcription, closed chromatin configurations are thought to repress gene expression. Repressive histone marks include H3K9me2 and H3K9me3, as wells as H3K27me3 [107].

Reprogramming takes place in a step-wise manner in a process involving a dynamic interplay between transcription factor binding, changes in genetic signatures, and changes in the chromatin landscape during the transitions [108–110]. A recent study by Suva and colleagues compares the epigenetic landscape of GBM stem-like cells to their differentiated counterparts and identified a subset of neurodevelopmental transcription factors sufficient to de-differentiate GBM cells to a stem-like state. Expression of these transcription factors (POU3F2, Sox2, SALL2, and OLIG2) was found to be sufficient to recapitulate the epigenetic landscape and phenotype of the original tumor initiating cell population, [111]. This study promotes the growing view that tumorigenesis results from both genetic and epigenetic changes [112]. The current dogma maintains that binding of transcription factors to DNA requires an open and accessible chromatin configuration, but recent studies indicate that core reprogramming factors Oct4, Sox2 and Klf4 are an exception to the rule. Soufi et al. [113] found that during the process of fibroblast de-differentiation to iPSCs, Oct4, Sox2, Klf4, and c-Myc (OSKM) can bind DNA in regions of closed chromatin configuration, allowing them to access their target genes very efficiently. The authors propose that these transcription factors are able to bind DNA in the context of the nucleosome by interacting with only one strand of the DNA helix. These findings may explain why these factors are so efficient at inducing de-differentiation of cells. During differentiation, lineage-specific genes are activated and pluripotency genes are inactivated via mechanisms that depend, at least in part, on chromatin state. If OSKM bind promoter regions of pluripotency genes in a closed chromatin configuration or induce changes in chromatin architecture to activate gene expression, these factors would be powerful drivers of de-differentiation.

As alluded to earlier, the balance between open and closed chromatin structures determines genome-wide gene expression states, and altering this balance can result in transcriptional deregulation that leads to disease (e.g. cancer) [114]. In fact, defects in histone methylation have been reported to play a role in tumor progression [115–119]. Studies found that mutations in EZH2 correlated with the onset of myelodysplastic tumors [115,116]. EZH2 encodes the catalytic subunit of polycomb repressive complex 2 (PRC2), the enzyme responsible for tri-methylation of histone 3 at lysine 27 (i.e. H3K27me3). Interestingly, EZH2 has been reported to enhance tumorigenicity by blocking differentiation of cancer cells in solid as well as hematopoietic tumors [120–122]. Furthermore, histone deacetylase (HDAC) inhibitors reduce stemness of multiple cancer cell types [123,124] and pharmacological inhibition of EZH2 has been found to induce apoptosis of cancer stem cells but not normal ES cells [125]. These indicate that histone modifications play a role in supporting the cancer stem cell phenotype and that therapeutic strategies focused on targeting epigenetic mechanisms could be a novel strategy to preferentially disrupt the cancer stem cell population.

MicroRNAs (miRNAs) are short non-coding RNAs that inhibit gene expression by targeting mRNA for degradation or by blocking translation of target genes [126]. These molecules control a wide range of biological processes and can function as both tumor suppressors and oncogenes as well as determinants of tumor cell stemness [127–131]. Expression of a defined set of miRNAs is sufficient to induce de-differentiation of human and mouse cells [132–134], highlighting that these factors can act to determine cell fate and suggesting an important role in CSC generation. In fact, expression of miR-302 is sufficient to reprogram skin cancer cells into a multipotent stem-like state. These cells not only re-expressed ES cell markers (e.g. Oct4, Sox2, Nanog, SSEA-3/4), but were also found to differentiate into different tissue cell types [129].

Given the potential implication in diagnosis and prognosis of cancer, several studies have focused on a cancer-specific miRNA signature [135] and, more recently, CSC-specific miRNA signature [136–139]. miRNA expression profiling in prostate cancer identified miR-34a to be under-expressed in the stem-like cell sub-population [136]. When re-introduced into tumor-initiating cells, miR-34a inhibited tumor growth and metastasis in vivo as well as sphere formation and migration in vitro. Although the authors did not comment on the ability of miR-34a to induce differentiation of these cells, other studies have shown that expression of this miRNA is sufficient to differentiate stem-like cells [140,141]. Similar to the effects of miR-34a in prostate cancer, forced expression of miR-let7a decreased cell proliferation, sphere-formation, tumor formation and metastasis of breast cancer tumor-initiating cells [137]. Differential miRNA expression has been also reported in glioblastoma (GBM) [142–144]. Interestingly, loss of miR-124 was found to enhance stemness and invasion of glioma cells [145] and miR-124 re-expression induced cell cycle arrest and differentiation of neuronal stem cells as well as tumor-derived stem cells [144]. These findings support the idea that a subset of miRNAs plays an important role in supporting the stem cell phenotype, not only in normal cells but also in tumor cells, and that targeting these factors could be a suitable way to deplete the tumor-initiating cell population and better manage cancer as a disease.

Reprogramming factors have been shown to promote expression of miRNA subsets in ES cells, implicating miRNAs in controlling ES cell identity [146]. Interestingly, reprogramming factors have been linked with miRNA regulation of oncogenic potential. For example, transcription factor c-Myc, one of the core reprogramming transcription factors, induces expression of miR-9 that primes breast cancer cells for epithelial to mesenchymal (EMT) transition and induces angiogenesis [147]. Myc can also inhibit the expression of tumor-suppressor miRNAs, resulting in increased cell survival of tumor cells [148]. Oct4, Sox2, and Nanog can induce expression of miR-302, which results in increased self-renewal and resistance of squamous cell carcinoma cells [149]. Conversely, miRNAs have been shown to regulate expression of reprogramming factors resulting in decreased tumorigenicity. For example, miR-145 inhibits lung cancer [150] as well as promotes differentiation of endometrial carcinoma cells by decreasing levels of Oct4 [151]. miR-7 can inhibit breast cancer metastasis to the brain, in part, by modulating Klf4 levels [152]. We recently described a novel molecular circuit by which the core reprogramming transcription factors Oct4 and Sox2 regulate stem-like phenotypes and tumor propagating capacity in glioblastoma through DNMT-dependent regulation of microRNA networks. We show that miRNA-148a is repressed by Oct4/Sox2 in a DNA methylation-dependent manner and this miRNA functions as an inhibitor of GBM cell stemness and tumor-initiating capacity [153]. Our study highlights a cross-talk between DNA methylation events, miRNAs, and reprogramming transcription factors that work together to regulate GBM tumorigenesis. These studies suggest a delicate interplay between miRNA expression and reprogramming transcription factors, and highlight how deregulation of either faction can result in expansion of the tumor-initiating cell compartment resulting in tumor formation and propagation.

## Concluding Remarks

There is a growing body of evidence that associates stem cell properties with the tumor-initiating cell population in human cancers. This CSC population is highly relevant to the biology of tumors and understanding their contribution to tumorigenesis holds great promise in terms of cancer treatment. Identifying and developing ways to target the tumor-initiating population will improve the way we treat cancer. One caveat of this approach is that CSCs and normal stem cells have similarities, therefore it is very important to understand the differences between normal stem cells and CSC in order to design cancer-specific therapies. With the growing number of studies looking at CSC-gene signatures, one can’t help but wonder if in the near future we will be able to merge these data to identify CSC-specific mechanisms that are common among all the cancers with the hopes of identifying a “primordial tumor-initiating network”. This “ideal” situation would provide us with potential “magic bullets” to treat cancer.

Our current knowledge of cell reprogramming mechanisms also opens the possibility that therapeutic approaches may inadvertently drive a subset of tumor cells toward a more stem-like and treatment-resistant state. This potential for “stress-induced de-differentiation” further highlights the importance of understanding the effects of current therapies not only when considering cytotoxicity, but also effects on de-differentiation and tumor-initiating potential. This hypothesis creates a new level of complexity and provides a plausible explanation for why tumors become resistant to therapy and recur. Understanding the specific microenvironments where these tumor-initiating cells reside will give us the option to target their niche as well as the specific cells themselves. Emerging data indicate there is a crosstalk between epigenetic modifications, miRNAs, and reprogramming transcription factors. Understanding the bidirectional regulation between these factors is becoming a new and interesting area of research. Identifying the components and circuitries that contribute to the generation of CSCs will allow us to design more rational therapies not only to target tumor-initiating cells, but to prevent them from appearing in the first place (e.g. cancer vaccines or therapies that block dedifferentiation). CSCs are extremely dynamic entities capable of adapting to different situations in order to maintain and propagate tumors, and although it appears that tumors arising in different tissues may have different cells of origin, one thing remains: understanding how transcription factor networks regulate tumor-initiating populations holds great potential for understanding tumor biology and advancing cancer therapy.
